# Perspectives of Nurses Toward Telehealth Efficacy and Quality of Health Care: Pilot Study

**DOI:** 10.2196/medinform.9080

**Published:** 2018-05-25

**Authors:** Ayisha Bashir, Dhundy R Bastola

**Affiliations:** ^1^ Department of Clinical and Translational Science Creighton University Omaha, NE United States; ^2^ School of Interdisciplinary Informatics, Information Science and Technology University of Nebraska Omaha, NE United States

**Keywords:** telehealth, survey, telemedicine, telenursing

## Abstract

**Background:**

Telehealth nursing, or the delivery, management, and coordination of nursing care services provided via telecommunications technology, is one of the methods of delivering health care to patients in the United States. It is important to assess the service quality of the involved health professionals as well as the telehealth nursing process. The focus of this study is the innovative model of telehealth care delivery by nurses for managing patients with chronic disease while they are living in their own residence.

**Objective:**

The primary objective of this pilot study was to examine whether telehealth technology impacts the perceived level of internal service quality delivered by nurses within a telehealth organization. To address this research goal, the notion of telehealth nursing service quality (TNSQ) is empirically tested and validated with a survey instrument.

**Methods:**

Data were collected from nurses belonging to a home care agency based on interview questions inquiring about facilitators and inhibitors to TNSQ. A survey to measure TNSQ based on the SERVQUAL instrument was completed by adjusting descriptions of the original instrument to suit the context. Follow-up interviews were conducted to validate questions on the revised instrument.

**Results:**

The findings of this survey research were positive, based on mean differences between expectations and perceptions of TNSQ. This indicates satisfaction with TNSQ and shows that the quality of the service is higher than what the respondents expect. The Wilcoxon signed-rank test using the *P* value for the test, which is .35, did not show a statistically significant change between the median differences of perception and expectation. The total number of respondents was 13. Results indicate that overall perceived service quality is a positive value (0.05332). This means the perceptions of the level of service are slightly higher than what they expect, indicating there is satisfaction with TNSQ.

**Conclusions:**

The responses to the interview questions and data gathered from the survey showed overall satisfaction with TNSQ. The SERVQUAL instrument was a good framework to assess TNSQ. In a nutshell, the study highlighted how the telehealth process provides daily monitoring of patient health, leading to the benefits of immediate feedback for patients, family, and caregivers as well as convenience of scheduling.

## Introduction

### Background

Telehealth encompasses all facets of remote health care including clinical services provided using telemedicine, as well as interactions with automated services, systems, or information resources. It is the integrated use of electronic information and telecommunications technology to support remote clinical health care, patient and professional health-related education, public health, and health administration [1]. The terms telehealth and telemedicine have become intertwined in literature; in this study, we use telehealth as a broad term that encompasses clinical and nonclinical services in contrast to telemedicine, which consists of purely clinical services [2]. Given the success of telehealth in specialty health care services, the use of technologies to transmit health information and provide care across a distance is poised to enter mainstream health service delivery [3,4]. Although many studies have assessed the cost-effectiveness and patient acceptance of this new method of service delivery, the perspective of nurses as the service provider has not been previously studied [5,6]. Thus, the aim of this study is to investigate telehealth nursing service quality (TNSQ) from the nurses’ perspective. To address this research goal, we developed and empirically tested the notion of TNSQ.

### Role of Nurses in Telehealth

One of the evolving roles of nurses as they take on new responsibilities of providing nursing service in home settings is the use of information technology (IT). Telehealth nursing focuses on patients’ long-term wellness, self-management, and health [1]. According to the American Telemedicine Association, this IT solution provides nursing care across a distance, empowering the care providers with the ability to monitor, educate, follow-up, collect data, and provide multidisciplinary care including remote interventions, pain management, and family support in an innovative fashion [7]. It has been reported that agencies using telehealth have an average patient-to-nurse ratio of 15:1, as compared with non-telehealth agencies having a ratio of 11:1 [7]. Therefore, telehealth nursing can make a tremendous difference in providing patient care, particularly in rural or underserved areas in states such as Nebraska, where there is generally a shortage of nurses and health care services, as well as resources can be limited [3]. In addition, in rural areas, many patients do not receive timely health care interventions because of the lack of specialist services [2]. Home health agencies with telehealth capability caring for patient populations with chronic diseases can take care of patients in their home setting and therefore fill this gap. This provides convenience and a sense of security to the patient, allowing timely nursing interventions under supervised physician care [2,7].

### Importance of the Study

This study is important and significant for several reasons. First, nurses’ perspectives toward telehealth service quality (SQ) is researched through a case study and validated with a survey instrument. Second, a positive role of telehealth nursing and related technology toward patient care is highlighted. Third, it establishes how telehealth provides daily monitoring of patient health, which has benefits of immediate feedback for patients, family, and caregivers as well as convenience of scheduling. Quality of service is essential for the telehealth performance and viability of the service; therefore, it is important to assess the SQ of involved health professionals as well as the telehealth nursing process [6,8].

### Service Quality

Among different characteristics of service, the quality of service in the health sector shapes the experience of care beyond technical competence. In the conceptual model of IT-SQ, it has been described as a comparison between levels of perceived services and consumer expectations [9], where the author suggests that SQ has a functional component concerned with *how the service is delivered* and a technical component concerned with *what is delivered*. This concept is implemented [10] by suggesting that SQ can be measured as the “gap” between the customers’ expectations of service and their perceptions related to the quality of service actually being delivered. In other words, SQ is a comparison of expectations (E) with perceptions (P) and can be represented as SQ=P−E [11]. This concept was further described and operationalized [9], leading to the development of the SERVQUAL instrument for SQ [10,11].

### Telehealth Nursing Service Quality

The conception of SQ has more to do with the “manner” in which employees deliver services to their customers, rather than the outcome of the service [11]. In a similar vein, TNSQ is the synthesis of the perceptions of the telehealth nurses from the functional and interactive aspects of telehealth interventions. In addition, the SQ items consisting of tangibles (TA), empathy (EM), assurance (AS), reliability (RL), and responsiveness (RS) are the dimensions measured by the SERVQUAL instrument [10]. Furthermore, these are applicable to the measurement of TNSQ [8,12]. In this study, the SERVQUAL instrument is modified and utilized to measure TNSQ [10] (Table 1). The SERVQUAL dimensions developed [10] and revised for this TNSQ study are described in Table 1.

Traditionally, the quality of nursing care was perceived to relate to the degree to which patients’ physical, psychosocial, and extra care needs were met [13]. The consequences of quality care were interpreted as *therapeutic effectiveness* where the therapy provided by nurses was perceived to positively affect patients’ healing. In other words, it referred to “how” the core service is delivered [14]. In health care services, expression of compassion and empathy by doctors or nurses is an example of functional quality, which also applies to telehealth nursing [13]. Telehealth nursing aims to propagate and advance the process of nursing quality care by enhancing the attributes of empathy, assurance, attention to detail, interpersonal and communication skills, responsiveness, and reliability, while adding convenience to the process [14,15]. Thus, telehealth nursing quality is described as a multidimensional construct that encompasses attributes such as empathy, assurance, and reliability.

**Table 1:** Adapting general service quality (SERVQUAL) dimensions to telehealth nursing service quality (TNSQ).

Serial number and dimension		General definition	TNSQ definition
1	Tangibles	The physical evidence of service	Physical facilities, equipment, and appearance
2	Reliability	The consistency of performance and dependability	The consistency of performance and dependability of telehealth nursing services
3	Responsiveness	The willingness and readiness of employees to provide service	The willingness and readiness of nurses to provide service
4	Assurance	The confidence communicated by the service provider	The confidence communicated by the telehealth nurse or provider
5	Empathy	The service provider’s efforts to understand the customer’s needs and then to individualize the service delivery	The nurse’s effort to understand the customer’s needs and then to individualize the service delivery

This study follows a research approach in which perspectives of telehealth nurses were elicited through a case study and administration of a survey (Multimedia Appendix 1). Expected results include a better understanding of nurses’ perspectives of telehealth interventions and quality of the health care services provided. Here, telehealth nursing quality is defined from the nurses’ perspectives as a *comparison of expectations (E) about telehealth nursing services with perceptions (P)* using the formula *TNSQ=P−E*. TNSQ attempts to capture nurses’ expectations and perceptions of telehealth nursing services as they impact patients.

### Service Quality and the SERVQUAL Instrument

As discussed previously, TNSQ is assessed using a modified version of the SERVQUAL instrument. The SQ encompasses 2 related components, namely, technical and functional quality [11]. The technical (core) component is defined as the “what” performed during the service. This concept is implemented [10] by suggesting that SQ has a functional component concerned with “how the service is delivered” and can be measured as the “gap” between the respondent’s expectations of service and their perceptions related to the quality of service being delivered [10,11]. To understand the gap between how the technological service is delivered and “what is delivered,” it is important to measure the SQ of different advanced information and communication technologies in various industries [16].

Previously, these SERVQUAL items have been used as a reliable and valid measure of SQ because of the dimensionality, item compositions, validity, and disconfirmation paradigm used in its measurement. The “type of technology” has a direct effect on barrier reduction, attitudes, and quality and quantity of communication [16]. There is a link between potential SQ facets and health care organizations’ adoption of health care information technologies and telehealth [17,18]. SERVQUAL and its improved versions are still widely used for measuring SQ in various sectors including health care, justifying SERVQUAL as an appropriate tool for TNSQ [6,12]. In the last 2 decades, the SERVQUAL instrument has been refined by the creators [10] to the useful acronym RATER: reliability, assurance, tangibles, empathy, and responsiveness [10,12]. The rationale behind the development of the general instrument is that although each service is unique in some aspect, there are other aspects that are applicable to all services in general [12,13]. This is also the case for evaluating SQ in telehealth. For example, in Greece, SERVQUAL was used to assess the quality services provided by the hospital, after the hospital was transferred to a new and modern location with up-to-date technology including telehealth services [12]. The study showed that the transfer to the new premises and new telehealth equipment substantially improved all of the 5 dimensions; however, the most improvement was seen in the “tangibles” dimension [12]. Other studies have been conducted in the health care industry using SERVQUAL instrument. The application of SERVQUAL instrument was beneficial in a study conducted to optimize the telemedicine model in Korea, ultimately improving the SQ of the telemedicine service providers by providing a checklist of the critical-to-quality telehealth processes, requirements, and expectations of the involved parties [6].

SQ is generally conceptualized in terms of 5 dimensions: tangibles, reliability, responsiveness, assurance, and empathy [10] (Table 1). One of the benefits of applying SERVQUAL for health care to telehealth nursing is that the 5 dimensions can be assessed overall as well as individually and the perspectives of nurses can be assessed thoroughly [6]. In a nutshell, through this research, nurses’ perceptions about their ability to deliver telehealth services to patients are collected.

### Telehealth Nursing Service Quality and the SERVQUAL Instrument

SERVQUAL remains a valuable tool to assess quality of health care services and assess the perceptions of the patients and health care providers related to new equipment and new hospital facilities [6,12]. The gap analysis method based on SERVQUAL has been used to calculate patient’s perceptions related to hospital SQ [19,20]. Published research is lacking with the instrument being used to measure nurses’ perspectives toward telehealth quality. This is what makes this pilot study unique, as past studies have been conducted to determine the quality attributes for telemedicine services from the perspective of the patients and physicians [18,19]. There is a need to further investigate the quality attributes for telemedicine services from the nurses’ perspectives, which have not been clearly defined before [5,8]. The TNSQ survey contains questions belonging to the 5 dimensions [10,12] (Multimedia Appendix 1). The gap method of subtracting nurses’ expectations from perceptions in each of the dimensions (using the survey) helps in assessing the TNSQ [10]. The dimensions having the negative gap score indicate that there is a barrier or an inhibitor present in that dimension; in the same manner, a positive value indicates that there is a facilitator in that dimension. This study addresses this gap in our understanding of telehealth care by developing concepts and empirically assessing TNSQ. In addition, the SQ items consisting of empathy, assurance, reliability, and responsiveness are dimensions measured by the SERVQUAL instrument [10]. These are reckoned to be applicable in measuring the TNSQ [6,10]. Further evaluation of the responses helps in indicating the positive factors, barriers, and facilitators of TNSQ and ties the responses to the interview question related to facilitators of telehealth barriers or inhibitors to nursing quality care [6,12].

## Methods

### Case Study Site

The Visiting Nurse Association (VNA) of Omaha was selected as the case study site because it employs telehealth interventions for patient care and because of its geographical proximity; in addition, the VNA telehealth nursing staff was willing to participate in the study. The organization’s website provides information regarding the telehealth home care process. After VNA agreed to participate in the study, preliminary interviews were conducted to understand its telehealth environment. To determine the extent of telehealth service provided by VNA and the characteristics of the participants receiving telehealth interventions (age of the patients and types of diseases being addressed), the deidentified patient data (no name or personal identifying values) were obtained from VNA. This consisted of the list of patients who were under VNA care or recently discharged in February 2016. These were all home care patients who were using telehealth services in their own residence. The median age of 205 patients was 74 years, including a total of 128 females and 77 males. The monitoring period of the patients is calculated from the day the patient starts receiving VNA telehealth services and ends on the date they are discharged from VNA. The median monitoring period of a patient was 25 days. The VNA data showed majority of these patients were suffering from chronic disease, were aged above 70 years, and were residing in a home care setting (for more than 3 weeks). This facilitated the research to determine the role of telehealth technology in helping elderly patients living with chronic disease in their home setting.

The methodology of conducting the TNSQ consisted of the following steps: (1) survey research, (2) preliminary interviews to understand the telehealth environment, (3) use SERVQUAL to measure TNSQ—adapted from the original instrument by adjusting the descriptions to suit the context, and (4) follow-up interviews to validate questions on the revised instrument.

### Survey Research for Telehealth Nursing Service Quality

The interview at VNA set the baseline for collection of quantitative analysis data. A questionnaire was designed based on SERVQUAL instrument and its 5 dimensions, namely, tangibles, reliability, responsiveness, assurance, and empathy [10]. Looking toward the domains of TNSQ, and bringing together the responses to the interview questions, the nurses had more concerns related to the equipment and its functionality (which belongs to the tangibles dimension). Further evaluation was added to the tangibles dimension in this study, relating to telehealth technology. These additions resulted in the increase in the number of questions from 21 to 25 (Multimedia Appendix 1). In addition, 3 open-ended questions were designed to gain insight toward the positive and negative perspectives of the nurses related to telehealth interventions. Therefore, the original instrument was revised by adjusting the descriptions to address the context.

### Measurement

As described above, the SERVQUAL instrument was used to measure TNSQ. The VNA approved the survey. A digital version of the survey was created using Survey Monkey, a Web based software tool that was suitable for our project. Survey Monkey is an online survey development cloud-based software as a service company, with headquarters in San Mateo California. The VNA’s chief executive nursing officer emailed the weblink to all participating nurses, which included all 13 nurses employed by the organization. Participants were queried about their expectations regarding quality of service with the incorporation of telehealth technology. Next, they were asked to rate their perceptions of the actual performance (or delivery) of service in the context of telehealth technology. Perceived levels of SQ with the use of telehealth technology were assessed based on the previous definition of SQ, which is obtained by the linear subtraction of service expectations (E) from perceptions (P) for each scale item, using a 7-point Likert scale, according to the SERVQUAL instrument [10]. The complete SQ instrument, which consists of a 25-item scale and accompanying instructions, is included in Multimedia Appendix 1. In this appendix, the first section lists the questions used to measure the nurses’ expectations of service in the context of telehealth SQ; the second section lists the items used in measuring their perceptions of the actual performance of service; and the third section includes the demographic questions and 3 open-ended questions. The modified SERVQUAL instrument consisted of 25 items, which included the 5 original dimensions. The first 7 questions belonged to the tangibles dimension (1-7), questions 8 to 12 belonged to the reliability dimension (8-12), questions 13 to 16 belonged to the responsiveness dimension (13-16), questions 17 to 20 belonged to the assurance dimension (17-20), and the last 5 questions belonged to the empathy dimension (21-25).

### General Perceptions of Visiting Nurse Association of Nebraska Telehealth Staff

The primary objective of this study was to examine whether telehealth technology impacts the perceived level of internal SQ delivered by nurses within a telehealth organization. Therefore, an in-depth interview was conducted with VNA staff to elicit and analyze the perspective of the telehealth nursing team. VNA services include companion care, infusion pharmacy, home care, home health technology, hospice, and palliative care. VNA telehealth nursing services include monitoring the patient through assessment and collecting data, heart rate, blood pressure, weight, oxygen saturation, and temperature.

**Figure 1:**
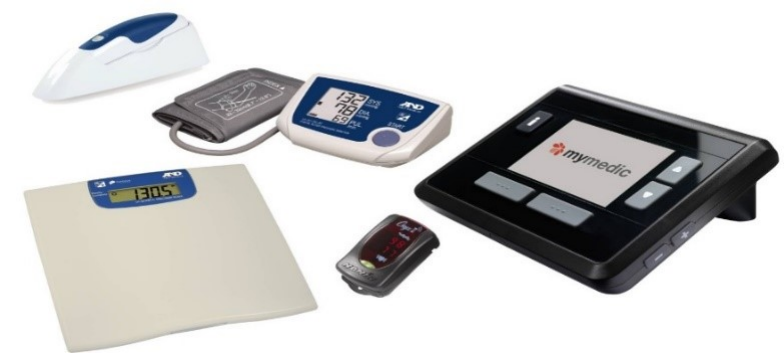
Telehealth system.

For the interview, the VNA telehealth team consisted of 3 members: the central station clinician, the clinical manager, and the chief operating officer. The proceedings of the interview were recorded with permission from all participants. The research questions were investigated during the interview, concentrating on the positive and negative aspects of telehealth. This team of nurses highlighted the process of telehealth patient care and daily monitoring. Every patient admitted to VNA services receives a disease-specific home telehealth kit and an educational session on using the equipment (Figure 1). Other members of the household or caregivers may monitor the health care process. The patients are triaged according to their vitals. The central station clinician is responsible for the initial interpretation of the data and contacts the patient with any health care concerns, including blood pressure changes, weight gain, or oxygen level fluctuations. They work closely with physicians, case managers, and other health care providers. The admitting nurse sets a plan with the patients, so they understand the process. One VNA case manager nurse monitors the patient, although different staff may monitor on weekends. Many patients are stable patients; however, others have urgent needs. The service is available 24 hours a day, 7 days a week. The current volume of patient load is manageable, and the maximum is 220 patients.

The below-mentioned interview questions addressed the factors that the nursing staff considers vital toward improving quality of patient care and whether telehealth played a role in providing this to patients. The staff shared perspectives on telehealth contributing and enhancing quality of care as well as barriers and inhibitors to providing quality nursing care via telehealth as described below:

1. What are the positive facilitators of telehealth contributing or enhancing the quality of care?
2. What are the telehealth barriers or inhibitors to nursing quality care?

The telehealth facilitators contributing and enhancing the quality of care mentioned by the VNA during the interview included quality of life and improved communication with health care providers, patients, and caregivers. Some of the telehealth nursing staff statements recorded during the interview include the following:

Telehealth interventions improve patient day to day living and enhanced the quality of life. The telehealth system is positive for the VNA as it emphasizes patient safety. It provides independence as well as a sense of security to the patient and caregivers, due to daily monitoring.

Telehealth improved communication with health care providers, patients and caregivers. This allowed clinicians to customize patient care quickly and effectively. As a result, the cardiologist and pulmonologist trust the VNA and this trust are gained over time due to their good work ethics and effective delivery of clinical services to the patients.

Telehealth interventions are helpful to patients, caregivers and for physician feedback, which leads to everyone’s ease of mind. During discussion with staff it was noted the organization’s desire to add electronic health records (EHR) and videoconferencing capability to their services. Their goal for adding videoconferencing is to improve timely communication by adding physician, patient, family and telehealth staff on board at the same time for consultation.

Telehealth nursing staff

## Results

### Data Analysis

This study discovered qualitative and quantitative results. A weblink for the survey was provided via email to all the VNA participant nurses. A total of 13 nurses responded to the survey. The survey was generated using a Web-based software tool, Survey Monkey. The data were downloaded from Survey Monkey and processed using SPSS version 22. Descriptive statistics of expectations, perceptions, and gap scores were calculated; the total number of respondents was 13, nonparametric and not normally distributed. To compare paired means for continuous data that are not normally distributed, the nonparametric Wilcoxon signed-rank test was performed. The result from these data is explained in the quantitative analysis and gap score analysis section (Tables 2-4).

### Qualitative Data Analysis

The VNA telehealth staff responses to the questions asked during the exploratory interview showed how the telehealth methods were helping the nurses to achieve good quality patient care, at the same time, providing peace of mind to the clients. These results were possible because of early interventions and monitoring. The telehealth team considered improved quality of life and peace of mind as the most important factors toward providing good quality patient care. The positive facilitators of telehealth, contributing or enhancing quality of care mentioned by VNA, were related to patient safety and quality of life and improved communication with health care providers, patients, and caregivers. In addition, TNSQ is achieving highest standards through the incorporation of technology and teaching patient’s personal health habits. Some of the barriers mentioned in the interview consisted of incompatibility and inconsistency of equipment in some cases. For example, the inability to add diabetic patients to their service was an issue at this time because of glucometer incompatibility in some patient’s equipment. The response to the open-ended questions in the survey matched the response to the interview questions related to facilitators and inhibitors, as shown by the dimension mapping (Multimedia Appendix 2).

### Quantitative Data Analysis

The survey instrument was approved by the University of Nebraska Medical Center (for the University of Nebraska at Omaha) institutional review board (IRB) for protection of human subjects, as it involved survey and interview of nursing professionals. It was qualified as exempt (IRB # 472-16-EX). The data collected from online survey (quantitative data), focus group case study, and interviews (qualitative data) were used to find existing perception of nurses regarding TNSQ. After approval, the surveys were administered through an online software tool to VNA participants as the VNA office was the case study site. The original SERVQUAL instrument was modified by adding technological aspect of the telehealth interventions to the tangible section of the questions. First, the respondents were asked the expectation of SQ because of the incorporation of telehealth technology. Next, the respondents were asked to provide perceptions of the actual performance (or delivery) of service because of telehealth technology. Then, the perceived levels of SQ because of the incorporation of technology were assessed by the linear subtraction of service expectation (E) from perception (P) for each scale item.

### Gap Scores Analysis

The distribution scale values obtained for the average telehealth service expectation, service performance, and SQ scores are presented in Tables 2-4. The gap scores are negative in the tangibles (1-7), responsiveness (13-16), and assurance dimensions (17-20). These values demonstrate that the expectations (E) of telehealth nursing service are higher than the actual perception (P) of the service (Table 3,Multimedia Appendix 1). The gap scores are positive in the reliability (8-12) and empathy (21-25) dimensions, which means that the performance in reliability is more than expectations. This has a positive effect on the level of the nursing services within the telehealth provider system based on the incorporation of telehealth nursing services. The values of the items scored on a scale of 1 to 7 can vary from a possible minimum of 25 (25×1) to a maximum of 175 (25×7). Therefore, the more positive the value, the higher the perceived telehealth service item (Table 2).

**Table 2:** Descriptive statistics.

Dimension	Minimum	Maximum	Mean (SD)	Skewness (SE)	Kurtosis (SE)
Tangibles	−0.90	0.40	−0.50 (0.50)	1.80 (0.845)	3.19 (1.74)
Reliability	0	0.80	0.395 (0.27)	0.275 (0.845)	2.16 (1.74)
Responsiveness	−0.70	0	−0.12 (0.26)	−1.33 (0.845)	2.16 (1.74)
Assurance	−0.90		−0.11 (0.36)	−2.21 (0.845)	4.92 (1.74)
Empathy	0	0.90	0.39 (0.36)	0.99 (0.845)	−0.31 (1.74)

**Table 3:** Descriptive statistics.

Statement	Number of statements (N)	Minimum	Maximum	Sum	Mean (SD)
Expectation	25	5.40	7.00	162.50	6.58 (0.415)
Perception	25	5.60	7.00	164.10	6.48 (0.43)
Valid N (listwise)	25	–	–	–	–

**Table 4:** Hypothesis test summary of Wilcoxon signed-rank test.

Serial number	Null hypothesis	Test	*P* value	Decision
1	The median of differences between expectation (E) and perception (P) equals 0	Related-samples Wilcoxon signed-rank test	.35	Retain the null hypothesis

The scale items and sum of average showing higher positive values indicate higher perceived telehealth service items (Table 3). These values show that the perception of performance of telehealth nursing service is overall more than the expected level of SQ. The research generated 13 pairs of ranked data, which are nonparametric, not normally distributed. To compare paired means for continuous data that are not normally distributed, and to compare paired means for ranked data, the nonparametric Wilcoxon signed-rank test is selected [21]. The Wilcoxon signed-rank test using the *P* value for the test, which is .353 (Table 4), did not show a statistically significant change between the median differences of perceptions (P) and expectations (E). Results indicate that the overall perceived SQ is a positive value (0.05332), which means the perceptions of the level of service is slightly higher than what they expect, indicating there is satisfaction with TNSQ.

The responses to the interview and data gathered from the survey showed overall satisfaction with TNSQ. Some issues of equipment malfunction and inconsistency in the telehealth equipment were pointed out by the respondents of the survey. The results of this case study are helpful in reinforcing the positive role of telehealth in impacting patient care and providing alternative solutions to the management of chronic disease. The VNA experience of implementing telehealth methods in the case of chronic patients suffering from chronic obstructive pulmonary disease (COPD) and heart disease is leading to better quality of life, as well as offering preventive services at a lower cost. Elderly patients suffering from chronic diseases, especially COPD and heart disease, are mostly benefiting from the telehealth TH interventions [3,22].

### Overall Perceived Telehealth Nursing Service Quality

Overall SQ is measured by obtaining an average gap score of the SERVQUAL dimensions [10]. As shown in Table 2, the overall average gap score for the SQ of telehealth nursing is 0.05332. In other words, the overall perceived SQ is a positive value (0.05332), which means the perceptions of the level of service are slightly higher than what they expect, indicating there is satisfaction with TNSQ. Overall average gap score for the SQ is calculated as (TA+RL+RN+AS+EM)/5=0.05332.

### Demographics and Open-Ended Questions

All the 13 respondents who completed the survey were female, and majority of them were aged 35 years or older. In addition, most of the respondents were seen to have earned a bachelor’s degree in nursing. The responses indicate that the nursing staff considered the convenience of scheduling and monitoring patients daily as a benefit of telehealth nursing. This was like the response of the focus group interview question related to the benefits and facilitators of telehealth interventions leading to peace of mind of the patients and the nursing staff. These responses belong to the tangible dimensions, highlighting the benefits of technology, like the findings documented by previous studies [3,23].

The second open-ended question was asked to get the nurses’ perspectives related to what they liked most about telehealth. Eight of the responses obtained mapped mainly to the reliability dimension and one each to the tangibles and responsiveness dimensions. This indicated that the nurses like the security of monitoring the patients daily, which adds to the convenience and immediate review of the information, adding validity to the previous open-ended question and interview questions. The final open-ended question was intended to get the perspective of what the nurses did not like about telehealth. A total of 9 participants responded, and majority of their responses mapped to the tangibles dimension and only one to empathy. The responses highlighted the nurses’ perspectives related to what they did not like about telehealth, and these responses were on the same pattern as the interview question part b, emphasizing the barriers or negative aspects of TNSQ. The responses mostly belonged to the tangibles dimension related to equipment malfunction, inconsistency of technology, and update issues (Multimedia Appendix 2). There are similar findings documented in previous studies [3,24].

## Discussion

### Principal Findings

The responses to the interview and data gathered from the survey showed overall satisfaction with TNSQ. Some issues of equipment malfunction and inconsistency in the telehealth equipment were pointed out by the respondents of the survey. The results of this case study are helpful in reinforcing the positive role of telehealth in impacting patient care and providing alternative solutions to the management of chronic disease. The VNA experience of implementing telehealth methods in the case of chronic patients suffering from COPD and heart disease is leading to better quality of life, as well as offering preventive services at a lower cost. Elderly patients suffering from chronic diseases, especially COPD and heart disease, are mostly benefiting from the TH interventions [3,22].

### Limitations

This research was an initial pilot study on TNSQ. Due to the limit in time and resources, other telehealth nursing organizations were not contacted; therefore, the sample size was restricted to 13 participants belonging to the same organization VNA.

### Implications for Future Research and Practice

Conducting nation-wide nursing telehealth quality service studies would have a much more significant impact on research and perceptions of nursing telehealth quality service. Future research of telehealth interventions and chronic disease management in elderly home care will be important. A bigger sample size would allow comprehensive statistical data analysis to be possible. Other telehealth nursing organizations that are willing to participate in the survey will be contacted. More data will be collected, and CHERRIES checklist for reporting the online survey will be used as in this case study [25]. The user experience of the consumers, patients, and health care providers will be another aspect to follow-up. To understand the positive implementation of telehealth nursing, it is important to dig deeply into the successful models implemented, and learn from the barriers and obstacles that other organizations are facing in relation to the telehealth nursing intervention methods [21]. Any researcher who may consider telehealth nursing for future research should consider taking this survey to a larger number of telehealth nursing professionals; this will give quantitative validity. Comparisons with other nations such as Norway in Europe, where telehealth implementation is successful, would be of interest for research purposes [26].

### Conclusions

SERVQUAL instrument was a good framework to assess TNSQ. The main advantage of SERVQUAL was the ability to estimate not only the overall level of satisfaction but also to identify the dimensions where perceptions transcended expectations (indicating performance excellence in the dimensions of reliability and empathy) and dimensions where the experience falls short of expectations (the dimensions of tangibles, assurance, and responsiveness; Multimedia Appendix 2). The interview responses and data gathered from the survey showed overall satisfaction with TNSQ.

## Appendix

Multimedia Appendix 1 The telehealth nursing service quality (TNSQ) survey instrument.

Multimedia Appendix 2 Responses to the open-ended questions.

## Abbreviations

AS: assurance
ATA: American Telemedicine Association
COPD: chronic obstructive pulmonary disease
EM: empathy
IT: information technology
RATER: Reliability, Assurance, Tangibles, Empathy, Reliability
RL: reliabilty
RS: responsiveness
SQ: service quality
TA: tangibles
TNSQ: telehealth nursing service quality
VNA: Visiting Nurse Association
