# Supplementary material for: Perspectives of Nurses Toward Telehealth Efficacy and Quality of Health Care: Pilot Study
Source: JMIR Med Inform. 2018 May 25;6(2):e35. doi: 10.2196/medinform.9080 (PMC5993972; doi:10.2196/medinform.9080)
Supplement: Multimedia Appendix 1 [file medinform_v6i2e35_app1.pdf]

Multimedia Appendix 1: Survey Instrument

Instructions and internal service quality scale items for employee expectations and perceptions.

Instructions:

This survey deals with your opinions of telehealth services. Our research interest is in highlighting the efficacy and quality of telehealth encounters from the nurse's perspective. If you are an employee for a telehealth service provider, your participation is essential into the success of this study. We would highly appreciate your help and need a few minutes of your time. The survey consists of three sections and most of the questions can be answered with a check mark or a brief phrase of two or three words. PLEASE BE ASSURED THAT YOUR RESPONSES WILL BE HELD IN STRICT CONFIDENCE AND ONLY USED TO SECURE KNOWLEDGE ABOUT TELEHEALTH NURSING AND ITS RELATIONSHIP WITH QUALITY SERVICE

Please note that your participation is entirely voluntary. You may refuse to answer specific questions and may discontinue participation at any time. Your cooperation and participation is appreciated. The accuracy of the study depends heavily upon your response and has a major impact in determining the efficacy and quality of telehealth services from a nurse's perspective.

If you have any questions regarding this study, please feel free to contact us via e-mail at abashir@unomaha.edu or phone at +1(402)554-4899.

Thanks in advance for your participation.

============Section Break==========

DIRECTIONS: From your perspective, please choose the extent to which you think telehealth nursing services should possess the features described by each statement below. If you strongly agree that telehealth nursing services should possess a certain feature, please click on the appropriate radio button. Similarly, if you strongly disagree, select the appropriate button. If your feelings are not strong select one of the numbers in the middle. There is no right or wrong answers – all we are interested in is a number that best shows your expectations about telehealth nursing services.
Please choose the best answer from the following scale:
1=Strongly disagree;2=mostly disagree;3=slightly disagree;4=neutral;5=slightly agree;6=mostly agree;7= strongly agree

SECTION I: This section deals with ***expectations*** of the nursing services within the telehealth provider system based on the incorporation of telehealth services. Please indicate the extent which you agree or disagree with the following statements.

As a telehealth nurse it is my expectation…

E1. I should have up-to-date telehealth equipment (e.g desktop computer, central station monitor).

E2. I should have telehealth equipment that consistently connects and functions as designed.

E3.I should have telehealth devices function as intended and provide features (e.g., biomarker monitoring) that contribute to patient care.

E4.I should be able to obtain necessary information needed to take appropriate action concerning patient care and facilitate diagnosis.

E5.I should be able to easily maneuver and navigate use of telehealth nursing software and equipment.

E6. I should be dressed professionally and appear neat, while providing telehealth services.

E7. I should be able to obtain support for using the telehealth equipment.

E8. When I promise to do something for my patients by a certain time, I should do so.

E9. When patients have problems, I should be sympathetic and reassuring.

E10. The telehealth service provider should be dependable.

E11. I should provide the telehealth services at the time I promise to do so.

E12. I should keep the patient records accurately.

E13. I shouldn't be expected to tell patients exactly when services will be performed.(-)

E14. It is not realistic for patients to expect prompt services from myself and other nursing staff.(-)

E15. My colleagues and I don't always have to be willing to help patients.(-)

E16. It is okay if other nurses and I are too busy to respond to patients requests promptly.(-)

E17. Patients should be able to trust all the nursing staff of the telehealth organization.

E18. Patients should be able to feel safe in their interactions with me and other nursing colleagues.

E19. I should be polite and professional in my verbal conversations.

E20. My colleagues and I should get adequate support from the telehealth provider to do our jobs well.

E21. I should not be expected to give patients individual attention. (-)

E22. My colleagues and I cannot be expected to give patients personal attention.(-)

E23. It is unrealistic to expect me to know all the needs of my patients.(-)

E24. It is unrealistic to expect my colleagues and I to have the patient's best interests at heart.(-)

E25. My colleagues and I shouldn't be expected to have operating hours convenient to all the patients.(-)

SECTION II: This section deals with your ***perceptions*** of the nursing services within the telehealth provider system based on the incorporation of telehealth technology. Please indicate the extent to which you agree or disagree with the following statements:

As a telehealth nurse it is my perception…

P1. Our telehealth provider has up-to-date equipment. (e.g. desktop computer, central station monitor)

P2. Our telehealth provider has equipment that consistently connects and functions as designed.

P3. The telehealth devices function as intended and provide features (e.g., biomarker monitoring) that contribute to patient care.

P4. I am able to obtain necessary information needed to take appropriate action concerning patient care and facilitate diagnosis.

P5. It is easy to maneuver and navigate use of telehealth nursing software and equipment.

P6. Our nurses are dressed professionally and appear neat, while providing telehealth services.

P7. We are able to obtain support for using the telehealth equipment.

P8. When I promise to do something by a certain time, I do so.

P9. When the patient has problems, my colleagues and I are sympathetic and reassuring.

P10. As a telehealth nurse I am dependable.

P11. I provide services to the patients, at the time I promise to do so.

P12. I keep my records accurately.

P13. I do not tell patients exactly when services will be performed.

P14. Patients do not receive prompt service from me and my fellow nursing staff.(-)

P15. I am not always willing to help patients.(-)

P16. My colleagues and I are too busy to respond to patients requests promptly.(-)

P17. Patients can trust me and my colleagues.

P18. Patients feel safe in interactions with me and other staff members.

P19. My colleagues and I are polite and professional in our verbal conversations.

P20. My colleagues and I get adequate support from the telehealth provider to do our jobs well.

P21. I do not give patients individual attention.(-)

P22. Staff of the telehealth organization does not give patients personal attention.(-)

P23. I do not know what the patient needs are.(-)

P24 My colleagues and I do not have patient's best interests at heart.(-)

P25. My colleagues and I do not have operating hours convenient to all the patients.(-)

SECTION III:

What is your gender?

○ Female

○ Male

What is your age?

○ 18 to 24

○ 25 to34

○ 35 to 49

○ 50 and older

What is your level of education?

○ Bachelor in Nursing

○ Masters in Nursing

○ Nurse practitioner

○ Other (please specify)

What type of organization do you work for?

○ Private

○ Not-for-profit

○ Government

○ Hospital

○ Other (please specify)

What category best describes your position within telehealth organization?

☐ Telehealth nursing staff
Central station clinician

☐ Clinic manager

☐ Chief operating officer

☐ Other (please specify)

How did Telehealth change the way you work?

What do you like most about telehealth?

What do you like least about telehealth?

DONE
