# Supplementary material for: Perspectives of Nurses Toward Telehealth Efficacy and Quality of Health Care: Pilot Study
Source: JMIR Med Inform. 2018 May 25;6(2):e35. doi: 10.2196/medinform.9080 (PMC5993972; doi:10.2196/medinform.9080)
Supplement: Multimedia Appendix 2 [file medinform_v6i2e35_app2.pdf]

Multimedia Appendix B: Responses to the open ended questions:

Table A: Response to open ended question "How did telehealth change the way you work?".

Respondent	Responses	Dimension Mapping
1	Scheduling and follow up visits, patient teaching tool	Tangibles
2	Allowed us to incorporate a new resource into what is offered in our care - and highlighted the benefits of technology	Tangibles
3	Able to monitor patients even on days a nurse is not visiting them	Tangibles
4	Aids in teaching clients personal health habits	Empathy
5	The change in practice has improved keeping patients out of the hospital.	Tangibles
6	Assists with monitoring vs and weight on non-nursing visit days	Reliability
7	Phone contacts and assessments can be just as valid as in person visits when you listen and ask the right questions	Responsiveness

Table B: Response to open-ended question "What do you like most about telehealth?"

Respondent	Responses	Dimension Mapping
1	The security it gives my patients, ability to monitor them on days I do not visit them.	Reliability
2	Convenience	Tangibles
3	Ability to see how a patient is doing every day, despite when visits are scheduled to the home	Reliability
4	We are able to check on patients every day.	Reliability
5	Immediate review of information	Responsiveness
6	Ability to watch patients on a daily basis.	Reliability
7	Daily records	Reliability
8	Ability to see how patients are doing daily not just at appointments	Reliability

Table C: Response to open-ended question "What do you like least about telehealth?"

Respondent	Responses	Dimension Mapping
1	Time consuming to look up history of VS, weights for those patients that do not manually record	Tangibles
2	Equipment malfunction	Tangibles
3	Technology can become obsolete and cost to replace or update can be quite high	Tangibles
4	I do not like when the monitors do not function properly	Tangibles
5	When it does not work	Tangibles
6	The older equipment (not the IA monitors) are not easily set up and//or as reliable as the IA monitors	Tangibles
7	Nothing at this point	Tangibles

8	Inconsistency with equipment	Tangibles
9	When staff or patients question the results when I know equipment is reliable and tested.	Empathy
